# Literature Review of Associations among Attributes of Reported Drinking Water Disease Outbreaks

**DOI:** 10.3390/ijerph13060527

**Published:** 2016-05-27

**Authors:** Grant Ligon, Jamie Bartram

**Affiliations:** The Water Institute and Department of Environmental Sciences and Engineering, Gillings School of Global Public Health, University of North Carolina at Chapel Hill, 135 Dauer Drive, Chapel Hill, NC 27599, USA; jbartram@email.unc.edu

**Keywords:** waterborne disease, outbreak, drinking water supply, water system deficiency, case count, attack rate

## Abstract

Waterborne disease outbreaks attributed to various pathogens and drinking water system characteristics have adversely affected public health worldwide throughout recorded history. Data from drinking water disease outbreak (DWDO) reports of widely varying breadth and depth were synthesized to investigate associations between outbreak attributes and human health impacts. Among 1519 outbreaks described in 475 sources identified during review of the primarily peer-reviewed, English language literature, most occurred in the U.S., the U.K. and Canada (in descending order). The outbreaks are most frequently associated with pathogens of unknown etiology, groundwater and untreated systems, and catchment realm-associated deficiencies (*i.e.*, contamination events). Relative frequencies of outbreaks by various attributes are comparable with those within other DWDO reviews, with water system size and treatment type likely driving most of the (often statistically-significant at *p* < 0.05) differences in outbreak frequency, case count and attack rate. Temporal analysis suggests that while implementation of surface (drinking) water management policies is associated with decreased disease burden, further strengthening of related policies is needed to address the remaining burden attributed to catchment and distribution realm-associated deficiencies and to groundwater viral and disinfection-only system outbreaks.

## 1. Introduction

Drinking water disease outbreaks (DWDOs) occur when bacterial, protozoan or viral pathogens or toxic chemicals contaminate drinking water ingested by humans. This type of disease transmission differs from water-washed, water-based and water-related diseases, whose transmission pathways involve inadequate personal hygiene, parasites living within intermediate aquatic organisms, or water-related insect vectors, respectively [1]. A waterborne (or foodborne) outbreak is defined by the World Health Organization (WHO) as when at least two people contract similar illnesses after consuming the same water (or food) and epidemiological analysis identifies the water (or food) as the origin of the illness [2]. Such outbreaks can be large, especially where urban populations receive water through a single water supply system. They are frequently associated with diarrheal illness (e.g., 403,000 estimated cases of cryptosporidiosis in the 1993 Milwaukee, U.S. outbreak [3]) and occasionally high levels of mortality (e.g., 8500 estimated deaths in the 1892 cholera outbreak in Hamburg, Germany [4]).

Awareness of the public health impact of compromised water supplies has evolved since John Snow’s analysis of and response to a cholera outbreak in London in 1854 [5], which played a major role in establishing the science of epidemiology and discipline of public health. Nevertheless, at the start of the 21st century, few countries conducted national waterborne disease outbreak surveillance (NWDOS) to identify and describe waterborne disease outbreaks (typically including DWDOs and outbreaks linked with recreational water exposure). Such surveillance could facilitate understanding of contributing factors and thus inform outbreak prevention. One example of NWDOS is the U.S. state reporting-based Waterborne Disease and Outbreak Surveillance System. The voluntary (thus likely incomplete) system was initiated in part by the U.S. Centers for Disease Control (CDC) in 1971 and transitioned into the electronic National Outbreak Reporting System (NORS) in 2009. The NORS has continued the production of semi-annual summaries (CDC Summaries, or CDCSs) [6] started in 1971. They include information related to: disease burden; water system size and source(s); treatment type; identified deficiency type(s) (system failure or other event associated with the outbreak); and causative pathogen(s).

According to the (U.S.) National Research Council, as of 2004, “most European countries do not have an adequate surveillance system for waterborne disease, while the situation in developing countries is even worse” [7] (p. 39). The 1997 WHO inquiry into the waterborne disease reporting practice of European countries from 1986 to 1996 found that half (26/52) of the countries returned the questionnaires and 36.5 percent of the countries reported their number of waterborne disease outbreaks, implying that the majority of those countries lacked the ability to report such data. Etiologic agent(s) or water system type details were available in 35.6 percent (277/778) of reported outbreaks, while information about basic treatment deficiencies or other outbreak linkage with water exposure was too sparse and not recorded in the WHO dataset [8,9]. While many countries such as the U.K., Sweden, France, Germany, Finland, The Netherlands, Denmark and Norway have implemented NWDOS or similar systems [10,11], DWDO surveillance data in Canada, where an NWDOS is not used [12], have been characterized as “erratic, not easily accessible and kept in diverse locations and formats” [13] (p. 254). This is potentially representative of, or better than, DWDO surveillance and reporting in much of the rest of the world.

Due to the lack of adequate DWDO surveillance and/or scarcity of standardized data on DWDO characteristics and contributing factors in much of the world [7,8,9,13], an opportunity is missed to enhance the understanding and prevention of drinking water-borne disease. This research increases that understanding by unifying heterogeneous DWDO literature through extensive literature review and synthesis of DWDO information, and by analyzing the attributes of and temporal trends in DWDOs. This provides evidence on the relative frequency of water system deficiencies and other outbreak attributes associated with high disease burden, and thereby can inform targeting of preventive water system improvements.

## 2. Materials and Methods

### 2.1. Search Strategy

The literature search was undertaken using the terms (waterborne disease* OR diarrhea OR gastrointestinal OR enteric) AND (human* OR people OR population*) AND (drinking water OR piped water OR tap water OR water supply*) AND (epidemic curve OR outbreak*). Four peer-review literature databases were searched between 18 July 2011 and 15 August 2011: PubMed, EMBASE, Web of Science (WoS) and Environmental Sciences and Pollution Management (ESPM). The citation lists of the sources subjected to full text review were also searched for additional sources, whose citation lists were also searched for additional sources (if subjected to full text review) and so on.

### 2.2. Inclusion Criteria

Sources retrieved from the four databases were selected for full text review if their abstract included (or indicated inclusion of) description of one or more DWDOs or pooled analyses of such outbreaks. Full texts published in English or with text that could be uploaded into Google Translate (*i.e.*, extracted from Portable Document Format [PDF] files) were subjected to review. During full text review, a source (and its outbreak(s)) was included if outbreak information was given regarding location (country), year (or decade), etiologic agent (either specified or described as unknown, *i.e.*, not ignored), description of associated system failure(s) and/or contamination event(s) (“deficiency”, either specified or described as unknown), and health data (estimated case count).

A source (and its outbreak(s)) was excluded if associated with food-borne, nosocomial or secondary transmission; if focus was primarily on the disease burden experienced by immunocompromised persons (with the case count attributed to healthy persons not being recorded); if it was hygiene- but not water contamination-associated (water-washed); if the implicated water was “not intended for drinking” (e.g., unprotected surface water not designated for consumption); if the identified cause was a chemical agent, *Naegleria fowleri* or *Legionella* species (*i.e.*, not communicable); or if the setting was a cruise ship or oil rig (“marine setting”), *i.e.*, where drinking water systems do not include the typical spatial realm of catchment.

### 2.3. Data Extraction

Data concerning key outbreak attributes (Table 1) were extracted from retained sources by the first author. When conflict among sources regarding data or classification by attribute was observed, the value or classification (e.g., case count) given by the majority of the sources for each outbreak was used. For example, the case count of the largest-recorded DWDO (the 1993 Milwaukee Cryptosporidium outbreak) was recorded as 403,000 given that was the case count cited in the majority of related sources, although another source put case count as low as 1% of that figure, implying that bias (and outliers) could be introduced when the majority of sources substantially overestimated DWDO disease burden [14]. Data extraction was also complicated by the lack of universal definitions for several potential attributes, e.g., the CDCS terminology of Community, Non-Community and Individual Water Systems (only used in the U.S.) and Strength of Association, which links outbreaks with drinking water based on various water quality, epidemiological, laboratory and other findings as shown for U.S. [15] and U.K. [16] outbreaks. Given how differing definitions hinder efficient comparison of identified outbreaks based on such attributes, this potentially valuable information was not recorded.

### 2.4. Data Analysis

Outbreaks were grouped by decade according to their year(s) of occurrence as: pre-1920 (1854–1919), 1920–1929 outbreaks as “1920s”, and so on up to “2000+” for outbreaks from 2000 to 2010. Outbreaks spanning two decades were grouped within the decade in which the majority of their months occurred and in the earlier decade when they equally spanned two decades. The same approach was used for classifying multi-year outbreaks by year.

The frequencies and total case counts for national and subnational outbreak groupings were weighted by population (per 100,000 persons), with population data for 1980 being used as that decade endpoint was nearest the median year of all identified outbreaks. Population data for U.S. states were obtained from US Census Bureau [18] and for the rest of the world from the United Nations Statistics Division [19].

Individual outbreak case count and attack rate were treated as dependent variables, influenced by outbreak attributes, *i.e.*, the independent variables (rows 1–5 of Table 1). Comparisons were made between DWDO groupings associated with various attributes (e.g., for deficiency realm: only-catchment realm-associated deficiency outbreaks *versus* only-treatment realm-associated deficiency outbreaks), when both had number of observations (*n*) ≥ 30, using the Wilcoxon–Mann–Whitney test for two independent samples within SAS version 9.4 (SAS Inc., Cary, NC, USA) to accommodate non-normal distributions. Significant differences at *p* < 0.05 (two-tails value, with continuity correction) were noted. When number of attribute groupings exceeded two, simple Bonferroni adjustment was performed to determine an adjusted lower *p*-value to correct for multiple testing [20]. The comparisons by attribute did not include outbreaks with unknown or multiple characterizations (e.g., multiple pathogen types and multiple deficiency realms).

Analysis of outliers was performed on the overall case count and attack rate distributions by determining inner and outer fence values (as 1.5 and three times the interquartile range, respectively, below the lower quartile value and above the upper quartile value [21]) and removing resulting mild and extreme outliers (outside the inner and outer fence values, respectively) before conducting basic statistical analysis on the two adjusted distributions. Outbreaks (using unadjusted data) were plotted by their year and log_10_ case count or attack rate to seek the existence of a temporal correlation (through evaluating coefficient of determination).

## 3. Results

### 3.1. Source Inclusion and Outbreak Attribution

**Figure 1:**
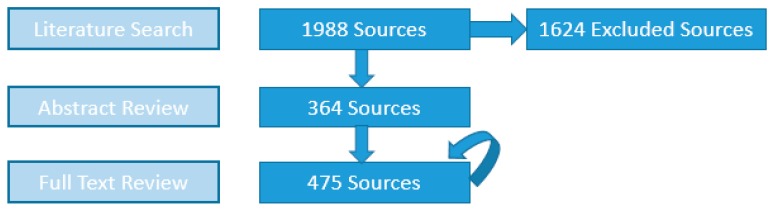
Source counts obtained after each of the three major stages of literature review (circular arrow represents addition of new full text-reviewed sources from reference lists of prior full text-reviewed sources).

Among the 1988 sources initially compiled from searching the four databases—545, 444, 254 and 745 elicited from PubMed, EMBASE, WoS and ESPM, respectively—the numbers of sources with abstracts containing DWDO information and those whose full texts subsequently fulfilled inclusion criteria for DWDO data extraction are shown in Figure 1.

Among all 475 sources, 417 (87.8%) contributed information for one to two outbreaks (usually in detail), 17 (3.6%) contributed information for three to nine outbreaks (often in tabular form), and the remaining 41 (8.6%) contributed information for at least 10 outbreaks via line-listing description (minimal detail within a table). The latter are best represented by the 24 CDCSs identifying 625 U.S. outbreaks from 1972 to 2008 and Gorman and Wolman [22] which identifies 416 U.S. and Canada outbreaks from 1920 to 1936; these together constitute 68.5% of all outbreaks identified. For the 1277 outbreaks first identified in the 41 sources that each describe at least 10 outbreaks (hereafter referred to as line-listing source outbreaks), the median case count and attack rate are 40 (*n* = 1277) and 24% (*n* = 127), whereas the median case count and attack rate for the other 242 outbreaks (non-line-listing source outbreaks) are 175 (*n* = 242) and 13% (*n* = 87), respectively. The line-listing source outbreak case counts are significantly lower than those of the non-line-listing source outbreaks (*p* < 0.0001), while the opposite is true for their attack rates (*p* = 0.0079).

### 3.2. Temporal, Geographic, Pathogen, Water Source and Treatment, and Deficiency Attributes of Outbreaks

Pooling all 1519 identified outbreaks, the mean, median and interquartile range for case count are 890, 49 and 18–187, respectively and the mean, median and interquartile range for percent attack rate (expressed in percent) are 27.4, 18.9, and 5.5–43.8, respectively (*n* = 214). For the overall case count distribution, with those lower and upper quartile values of 18 and 187, only upper inner and outer fence values of 440.5 and 694 can be obtained (implying upper mild and extreme outlier values of ≥441 and ≥695, respectively). With mild outliers removed, the adjusted case count distribution (*n* = 1291) mean, median and interquartile range are 73, 35 and 15–91, respectively, while with extreme outliers removed, the adjusted case count distribution (*n* = 1342) mean, median and interquartile range are 90, 37 and 16–105.75, respectively. For the overall attack rate distribution, with those lower and upper quartile values of 5.5% and 43.8%, respectively, no logical fence or outlier values can be established.

Basic statistics for outbreak attribute groupings are presented in Table 2, Table 3, Table 4, Table 5, Table 6, Table 7, Table 8 and Table 9 (in each Table, “*n*” signifies the number of outbreaks from which the calculated statistic is derived). For outbreak attribute groupings with *n* ≥ 10, means are 1.1–88.1 and 0.9–33.8 times greater their medians (for case count and attack rate, respectively). Standard deviations are relatively high at 0.9–15.2 and 0.5–1.3 times their means (for case count and attack rate, respectively).

**Figure 2:**
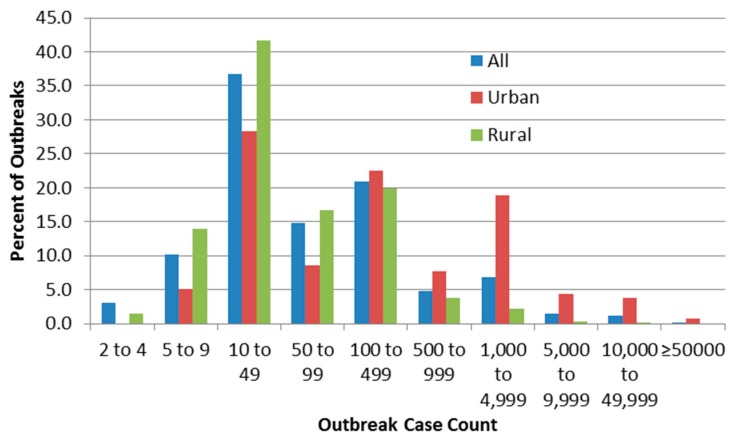
Distribution of outbreaks by case count.

Figure 2 shows the relative frequency of identified outbreaks (percent of all, urban or rural outbreaks) by case count based on urban/rural status.

#### 3.2.1. Temporal Associations

**Figure A1:**
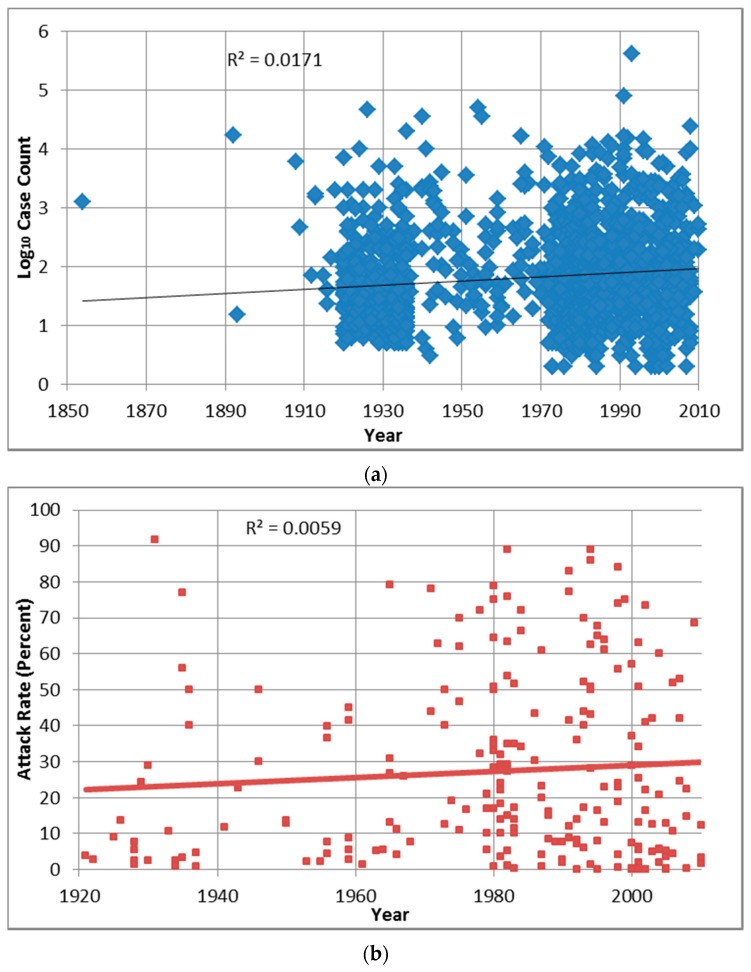
Distribution and correlation of outbreak log_10_ case count (**a**) and attack rate (**b**) with year.

Outbreak attack rates for the 2000+ decade (median in Table 2) are almost significantly lower than 1980s and 1990s outbreak attack rates (*p* = 0.0043 and 0.0034, respectively, are greater than the Bonferroni adjusted *p* of 0.0011). The relationships between log_10_ case count or attack rate and year indicate that the data (unadjusted for any potentially confounding factors such as demographics) do not fit any statistical model, with the coefficient of determination (R^2^) for each comparison of 0.02 and 0.01, respectively (Figure A1).

#### 3.2.2. Geographic Associations

Among the nine countries with at least 10 identified outbreaks (Table 3), India outbreak case counts are significantly higher than those of U.S., U.K. and Canada outbreaks (*p* < 0.0001 for all three comparisons are lower than the Bonferroni adjusted *p* of 0.0014). Outbreaks within developing countries (*i.e.*, those classified by the United Nations as having Human Development Index values <0.8 for 2014 [23]) comprise 5.8% (88 of 1519) of those identified, and have median case count (*n* = 88) and attack rate (*n* = 32) of 191% and 7.9%, respectively. Outbreaks within developed countries have median case count (*n* = 1431) and attack rate (*n* = 182) of 46% and 23.6%, respectively. Developing country outbreak case counts and attack rates are significantly higher and lower than those in developed countries (*p* < 0.0001 and 0.0006, respectively).

Among the nine highest-recording case count US states (Table 4) with *n* ≥ 30, case counts for Florida outbreaks are significantly lower than those of other low median case count, high-recording states (e.g., compared with Colorado, Pennsylvania and Indiana outbreak case counts, with *p* = 0.0001, <0.0001 and 0.0005, respectively (lower than the Bonferroni adjusted *p* of 0.0014)). Statistical analysis was not performed on attack rates for outbreaks grouped for each of the nine states as only one of the states has *n* ≥ 10.

Rural outbreak case counts (Table 5) are significantly lower than urban outbreak case counts (*p* < 0.0001 is lower than the Bonferroni adjusted *p* of 0.0167). Urban outbreak attack rates are significantly lower than rural outbreak attack rates (*p* < 0.0001 is lower than the Bonferroni adjusted *p* of 0.0167).

#### 3.2.3. Associated Pathogens

**Figure 3:**
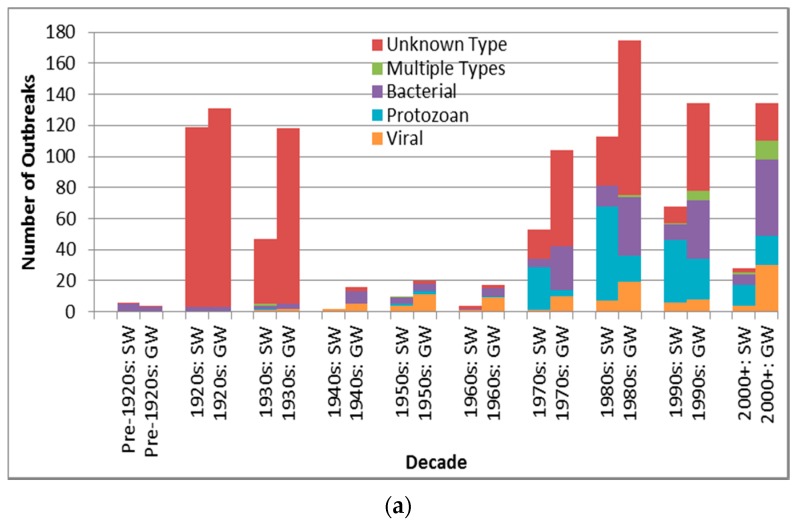

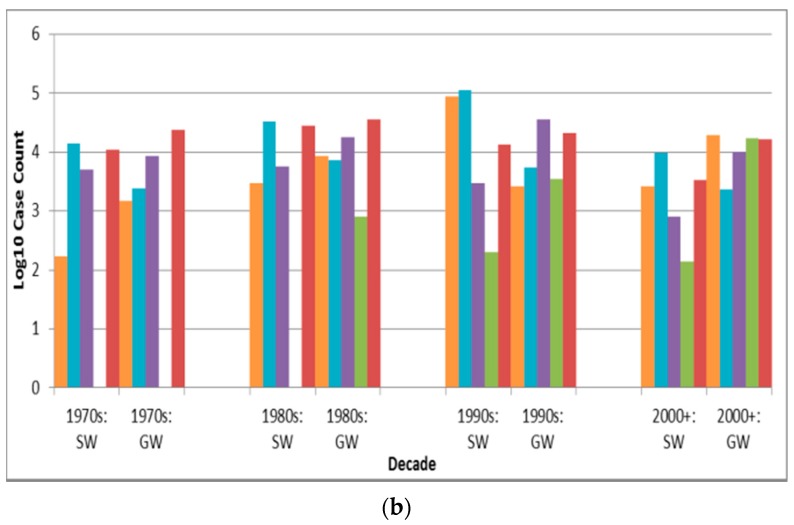
Pathogen type outbreak frequency for all recorded decades (**a**) and log_10_ decadal case count for 1970s–2000+; (**b**) by source type (SW is surface water, GW is groundwater).

Case counts for viral-associated outbreaks are almost significantly higher than those for protozoan-associated outbreaks (*p* = 0.0090 is greater than the Bonferroni adjusted *p* of 0.0050) (Table 6). Depiction of outbreak frequency and case count by decade, source type and pathogen type suggests potential temporal trends in occurrence and health burden for each pathogen type (Figure 3).

#### 3.2.4. Associated Water Sources

Surface water outbreak case counts are significantly higher than those of groundwater outbreaks (*p* = 0.0021 is lower than the Bonferroni adjusted *p* of 0.0083) (Table 7).

#### 3.2.5. Associated Water Treatment

**Figure 4:**
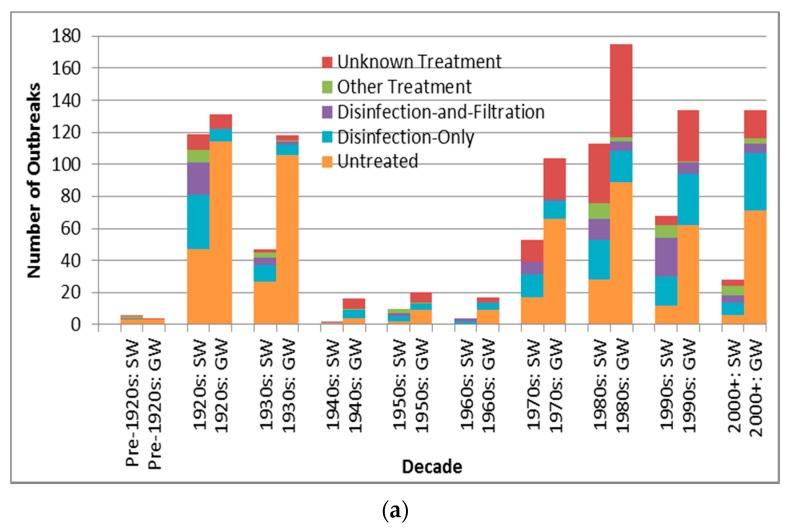

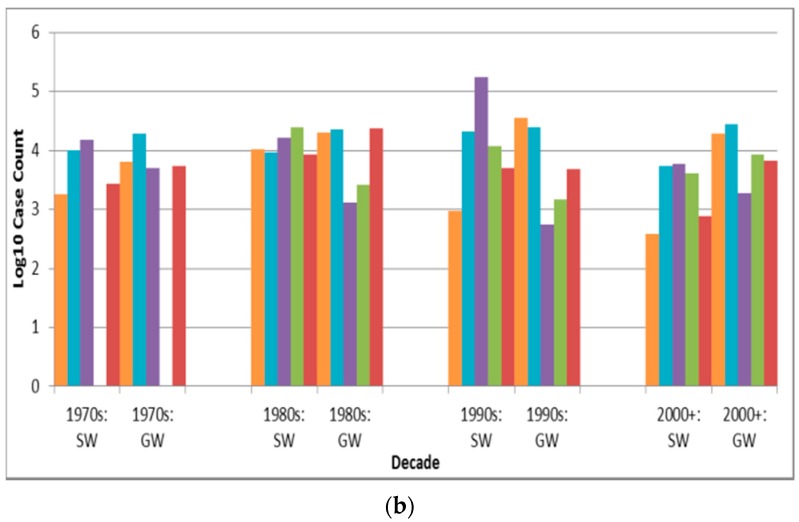
Treatment type outbreak frequency for all recorded decades (**a**) and log_10_ decadal case count for 1970s–2000+; (**b**) by source type (SW is surface water, GW is groundwater).

Case counts associated with untreated source outbreaks (Table 8) are significantly lower than those associated with disinfection-only and disinfection-and-filtration source outbreaks (*p* < 0.0001 for both comparisons are lower than the Bonferroni adjusted *p* of 0.0050). Depiction of outbreak frequency and case count by decade, source type and treatment type suggests potential temporal trends in occurrence and health burden for each treatment type (Figure 4).

#### 3.2.6. Associated Deficiencies

**Figure 5:**
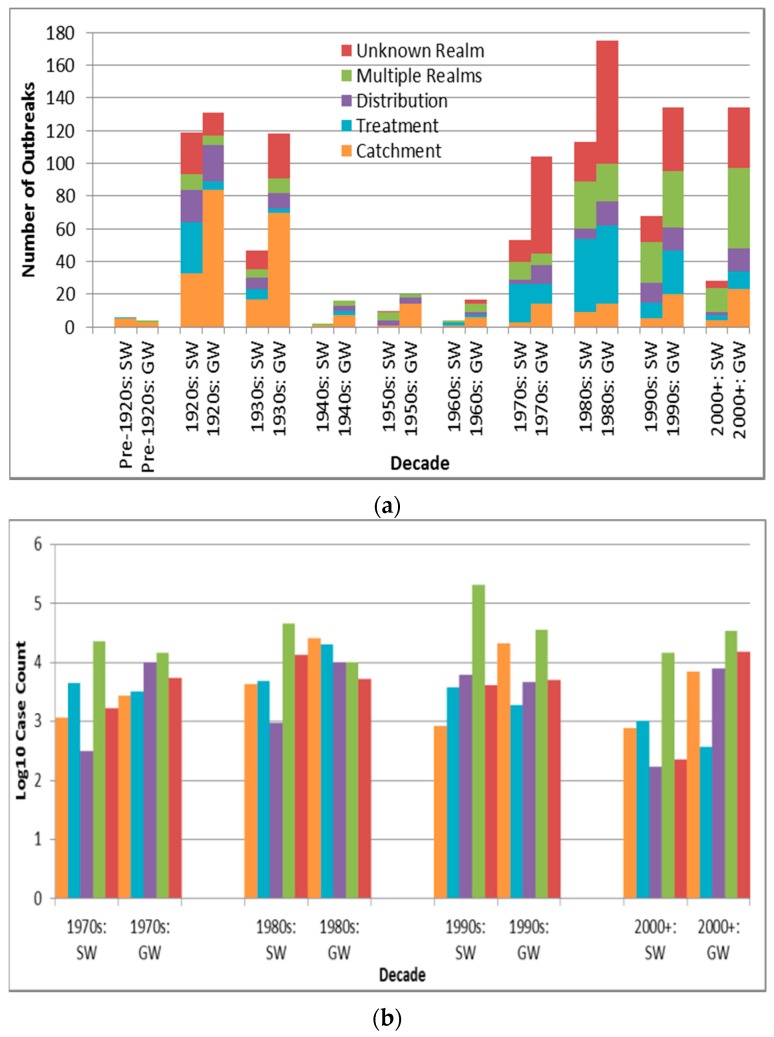
Deficiency realm outbreak frequency for all recorded decades (**a**) and log_10_ decadal case count for 1970s–2000+; (**b**) by source type (SW is surface water, GW is groundwater).

Among the major three deficiency realms (catchment, treatment and distribution, with deficiency types also described in Table 9), outbreaks associated with distribution realm-associated deficiencies have significantly higher case counts than outbreaks associated with deficiencies in the realms of catchment and treatment (*p* < 0.0001 and 0.0007, respectively, are lower than the Bonferroni adjusted *p* of 0.0024). Depiction of outbreak frequency and case count by decade, source type and deficiency realm suggests potential temporal trends in occurrence and health burden for each deficiency realm (Figure 5, with “Unknown Realm” including “Unknown”, “Untreated Surface Water” and “Untreated Groundwater” deficiency-associated outbreaks).

## 4. Discussion

### 4.1. Source Inclusion and Outbreak Attribution

While the majority (87.7%) of sources document one or two outbreaks, a small portion (3.6%) describing at least 10 outbreaks (referred to as line-listing sources) contribute the majority (84.1%) of outbreaks. This is especially true for the 25 line-listing sources of two types—the review by Gorman and Wolman [22] and the CDCSs, representing U.S. NWDOS data for 1920–1936 and 1972–2008, respectively. Together these contribute 68.5% of all outbreaks. The shallow depth in reporting in those reviews, which were the only source for 90.6% (943 of 1041) of the outbreaks described in them, characterizes the limited nature of reporting for the majority of identified outbreaks and indicates a major shortcoming of long-established yet voluntary U.S. NWDOS.

The finding that line-listing source outbreak case counts are significantly lower (*p* < 0.0001) than those of the non-line-listing source outbreaks, while the opposite is true for their attack rates (*p* = 0.0079), is consistent with the non-line-listing source outbreaks’ higher likelihood of being characterized as urban (51.2%, or 124 of 242 outbreaks) relative to line-listing source outbreaks (22.9%, or 293 of 1277 outbreaks). The more urban nature of the outbreaks associated with non-line-listing sources is logical in that such sources are the major contributor of outbreak data in jurisdictions lacking strong DWDO surveillance and reporting systems (which are more likely to produce line-listing type summaries), in which resources are more likely to be concentrated on often higher-profile, larger-scale urban outbreaks. Those outbreak case counts are higher than case counts of outbreaks from line-listing sources because urban water systems typically serve greater populations, increasing the potential for higher case counts. The fact that the opposite is true for attack rates is also consistent with the difference between urban and rural outbreak data as described in Section 4.2.2.

The heavy reliance on line-item CDCS reporting led to the general limitation cited in another multi-country DWDO review (of U.S. and Canada small drinking water system outbreaks from 1970 to 2014), in which “analysis of the data was limited by the large amount of missing data in many of the outbreak reports” [12]. One important example of inadequate outbreak characterization concerns the water system-supplied or associated-community population to be used as the denominator in deriving attack rate. Such data are available for only 14.1% and 27.7% of all identified outbreaks, respectively, which restricts the potential for determining the statistical significance of differences in attack rates. Given such shortcomings (and the prevalence) of simple line-listing of outbreak data in developed countries such as the U.S., and lack of such basic data gathering and public reporting in many developing countries, it is suggested that DWDO-reporting bodies worldwide adopt an alternative, standardized form of line-listing encompassing data categories such as those catalogued in this paper and beyond (e.g., site type, community or other source type, supply or community attack rate, strength-of-association with waterborne transmission, method and certainty in case count estimation, stool or other symptom count, nature of frequently multiple causative deficiencies, *etc.*). Standardizing and expanding upon the line-listing model currently used in several developed countries, rather than creating a more narrative-type model, would not only require minimal adjustment to current practice in those countries, but, more importantly, it would also provide a replicable template for jurisdictions without any public DWDO reporting system. This would facilitate public reporting of DWDO information, e.g., in peer-reviewed or online summary literature, and subsequent uptake by research such as this paper (for which requiring online data publication might have filtered ample DWDO information from developing countries out of this literature review, with their relative scarcity of data described in Section 4.2.2).

### 4.2. Temporal, Geographic, Pathogen, Water Source and Treatment, and Deficiency Attributes of Outbreaks

While the overall attack rate distribution does not contain mild or extreme outliers, that is not the case for the overall case count distribution, for which the removal of 228 mild or 198 extreme outliers greatly decreases the distribution’s mean, median and interquartile range lower and upper values (from 890, 49, 18 and 187 to 73–90, 35–37, 15–16 and 91–105.75, respectively). Using the adjusted distributions would decrease the influence of potentially improperly estimated case counts for many outbreaks (e.g., as described for the Milwaukee outbreak’s 403,000 cases of cryptosporidiosis in Section 2.3). However, given the lack of consensus in judging the validity of high case count estimates (and subsequent removal of related outbreaks from statistical analysis) and of relatively low case count upper thresholds (e.g., the 440.5 and 694 values for the overall case count distribution) in the scientific literature, the outliers were retained throughout the rest of the analysis.

Figure 2 suggests high frequency of reporting at case counts of 10–49 and 100–499, which could be due to authors (including those of the line-listing reviews) rounding numbers of cases to 10 or 100. The figure also confirms the logical inference that rural outbreaks are associated with low case counts compared to urban ones. The very low numbers of detected outbreaks with low case counts (below 10 cases) implies that outbreak surveillance may be ineffective in detecting smaller outbreaks which in fact may be more frequent than larger ones, or that reporting systems often round up those case counts to 10. In order to compare this threshold with one described elsewhere (by Craun, who stated thatmost outbreaks are identified only if at least one percent of the community population falls ill [24]), it would be necessary to obtain data on outbreak community population that were not present in most outbreak sources.

#### 4.2.1. Temporal Associations

It is likely that the attribution of 92.5% of identified outbreaks to the 1920s, 1930s, 1970s, 1980s, 1990s and 2000+ decades (with those decade groups each containing between 11.7% and 21.3% of all identified outbreaks) derives from the extensive line-listing reporting in Gorman and Wolman [22] and the 24 U.S. CDCSs rather than any changes in outbreak occurrence. Decadal outbreak frequency statistics contrast with decadal (total) outbreak case count (Table 2). Every decade except the 1990s contains at most 8.4% of identified cases, with the majority of the 1990s total case count of 641,243 cases (47.5% of the total) driven by the 403,000 cases in the 1993 Milwaukee Cryptosporidium (outlier) outbreak. Given how outliers can skew the total case count statistics—confirmed in the 1990s surface water protozoan, disinfection-and-filtration, and multiple deficiency realm bars (Figure 3b, Figure 4b and Figure 5b), respectively—total case count (the product of outbreak mean case count and frequency) is often less conducive to insightful analysis of outbreak attribute differences than is outbreak frequency, and is therefore not included in Table 5, Table 6, Table 7, Table 8 and Table 9.

#### 4.2.2. Geographic Associations

While the preponderance of U.S. outbreaks is evident (70.4% of all identified outbreaks), population-weighted outbreak frequency data (Table 3) suggest that Canada, the U.K., Sweden and Finland have similarly effective DWDO reporting systems. This is further supported by Finland and Sweden recording the highest and third-highest population-weighted total case counts among the nine countries assessed, respectively. However, although Sweden revamped its system in 1980, resulting in increased DWDO detection [25], that source’s figure of 71 outbreaks recorded in Sweden from 1986 to 1996 contrasts with the figure of 53 reported by that country (combining their number of DWDOs and recreational water outbreaks) to the WHO for the same period [9]. This inconsistency, potentially because Sweden’s “reporting and investigation system can be quite complex with a large number of different bodies being involved” [25] (p. 116) and thus might be conducive to conflicting reporting by differing agencies, which signals the need for enhanced reporting that likely applies to many other countries with non- or under-standardized systems.

The finding that India outbreak case counts are significantly higher than those of U.S., U.K. and Canada outbreaks (*p* < 0.0001 for all three comparisons), while India outbreak attack rates are lower than the attack rates of those three countries’ outbreaks (significance not tested due to *n* < 30 for India), is consistent with the nature of the significant differences between developing and developed country outbreak case counts (*p* < 0.0001) and attack rates (*p* = 0.0006). This could reflect how outbreak surveillance and reporting systems are likely stronger in the usually higher-resource developed country settings and are thus more able to detect and publish findings of (smaller) outbreaks outside major cities. The much more urban nature of the developing country outbreaks (57 out of 88, or 66%) compared to developed country ones (360 out of 1431, or 25%) implies that the former are likelier to have greater supplied populations potentially affected by the outbreaks, driving down attack rates.

Given that 42% of the world’s population (disproportionately located in developing countries) lacks piped-on-premises water and relies on supply types like public taps, unprotected dug wells and manually-collected surface water [26] that are less-represented in peer-reviewed DWDO literature, it is not surprising that only 5.8% of identified outbreaks are attributed to such countries. This underreporting is also likely to be associated with developing countries’ aforementioned likely weaker NWDOS systems (relative to those in developed countries), reasoning parallel to that in a review of waterborne disease on ships which concluded that “the number of reported outbreaks...is likely to be a small fraction of the total” (p. 437) due to lack of scientific literature publication and reporting to relevant authorities [27]. Combined with the likelihood of low probability of detection of low case count outbreaks, these factors imply that, for much of the world, collated drinking water disease data greatly underestimate the actual incidence of outbreak and endemic waterborne disease, even in actively-reporting countries [9].

Among U.S. states, Colorado and Oregon have the highest population-weighted outbreak frequency and total case count, respectively. This illustrates how outbreak data are related to both DWDO reporting system effectiveness—an association seen in Colorado, which received federal funding for reporting during 1980–1983 and saw an increase in outbreaks reported per year from 2.0 for the period 1971–1979 to 4.5 per year over those four years [28]—and underlying outbreak occurrence (greatly influenced by outlier events like the 1954–1955 Oregon outbreak with 50,000 estimated cases of giardiasis and the 1993 Milwaukee outbreak with 403,000 estimated cases of cryptosporidiosis). This strength in DWDO reporting for Colorado is also reflected in the low case counts for their outbreaks (likely arising from superior detection of small outbreaks) relative to those of outbreaks for other top-recording states (albeit with almost significantly greater case counts based on Bonferroni-adjusted *p* of 0.0014). The significantly lower case counts for Florida outbreaks relative to those of other states (e.g., for Colorado, Pennsylvania and Indiana, *p* = 0.0001, < 0.0001 and 0.0005, respectively) also imply the strength in DWDO reporting for Florida. This is further suggested by the case of the Florida Department of Health which has developed a Food and Waterborne Disease Program task force to assist county health departments and since 1997 has published annual outbreak reports linked to an online database [29].

The case counts of urban outbreaks are significantly (*p* < 0.0001) higher than the case counts of rural outbreaks. This is logical in that urban water systems typically serve greater populations and thus have greater potential for high case counts. However, while urban outbreak case counts are significantly higher than rural outbreak case counts, the opposite is true for their outbreak attack rates (with the difference significant, *p* < 0.0001).

#### 4.2.3. Associated Pathogens

Source authors attributed dozens of outbreaks to pathogens not normally associated with DWDOs, e.g., *Clostridium perfringens* and other indicator-type pathogens. With the aim of following the original text as closely as possible, such pathogen attributions were retained. Similarly, reported causative pathogen attributions were also retained for outbreaks lacking microbiological detection of pathogens in water or affected cases. Such outbreaks might also have been erroneously noted as single-pathogen rather than multiple-pathogen, especially in the case of sewage contamination-associated outbreaks, in which the fecal loadings likely contained a variety of fecal-oral pathogens. These types of pathogen attributions have less validity than those in sources in which the authors conservatively declared the pathogen as suspected or unknown types.

Figure 3a suggests a marked decline in unknown type etiology attribution (for the countries implicated in identified outbreaks) since the 1920s–1930s continuing through the 1980s to the 2000s, likely due to increasing ability to diagnose pathogens, for example, with the development of methods for Norovirus starting in the 1970s [30] and advent of Polymerase Chain Reaction testing. The relatively high overall unknown etiology proportion (53.3% of all identified outbreaks) corresponds well with the 54.9% proportion found in another recent multi-country DWDO review (of U.S. and Canada small system DWDOs from 1970 to 2014) [12], and contrasts the proportion found in a third multi-country review of DWDOs [11] in four Nordic countries (from 1998 to 2012). The relatively low unknown etiology proportion (29.7%) of the latter review is logical in that its authors attributed this to “improvements over time in methods and routines for microbiological analysis” (p. 8) that would better facilitate pathogen attribution during the review’s relatively recent time period of analysis. The potential strength and depth in reporting of NWDOS for those Nordic countries (relative to the voluntary, line-listing reporting-based US system) could also facilitate greater outbreak pathogen diagnosis and documentation, further supporting that low unknown etiology proportion.

Figure 3a also suggests a decreasing frequency of surface water protozoan-associated outbreaks, which corroborates the nearly statistically-significant (*p* = 0.0560) decrease in the annual proportion of U.S. surface water protozoan-associated outbreaks after 1989 as described in a 2010 review of 1971–2006 CDCS data [31]. This may largely derive from the increased oversight of surface water especially following recognition of waterborne transmission of *Giardia* and *Cryptosporidium* as exemplified by the passage of the 1989 U.S. Surface Water Treatment Rule (SWTR). The emphasis in the rule on those two protozoans, which are used for developing SWTR-specified treatment and monitoring programs, may also have contributed to the decreasing log_10_ decadal case count for surface water protozoan-associated outbreaks starting in the 1990s (Figure 3b). Decreasing log_10_ decadal case count for surface water outbreaks associated with each of the other four pathogen types from the 1990s might also derive from the implementation of the European Union (EU) Council Directive 75/440/EEC of 16 June 1975 Concerning the Quality Required of Surface Water Intended for the Abstraction of Drinking Water in Member States [32].

In contrast with the decrease in surface water viral outbreak frequency and log_10_ decadal case count starting in the 1990s, groundwater viral outbreak frequency and log_10_ decadal case count increased from the 1970s-onward. This could be due to slow progress in improving groundwater management policies (e.g., the delay in passage of the U.S. Groundwater Rule until 2007 [33]).

#### 4.2.4. Associated Water Sources

The finding that groundwater outbreaks are 1.9 times as frequent as surface water ones is comparable with proportions reported in two similar reviews of 3.2 [11] and 4.5 [12]. The latter may be elevated by the Pons *et al.* study’s focus on small systems (more likely to be rural than large systems given their association with populations of less than 5000 people in that study) if, as seems credible, a higher proportion of small systems are groundwater-fed. This is supported by comparing the rural-only outbreaks and urban-only outbreaks in the present review; the rural-associated proportion of groundwater to surface water outbreaks of 2.5 (390/159) is greater than the urban-associated proportion of 1.1 (184/162).

The higher prevalence of groundwater outbreaks relative to surface water ones, and of well outbreaks relative to those of the second-highest recording source subtype (rivers), might arise from groundwater sources being more vulnerable to contamination (because they often have fewer treatment steps) than surface water sources in the countries represented by identified outbreaks. However, this reasoning is called into question when considering how surface water sources can be more directly contaminated by human and animal fecal and other waste, and is confounded by the unknown number of groundwater sources studied in this review’s outbreaks that were under the direct influence of surface water. Surface water outbreaks having significantly higher case counts than groundwater outbreaks (*p* = 0.0021) could derive from how the former source type typically serves larger populations.

#### 4.2.5. Associated Water Treatment

The attribution of 46.3% of outbreaks to untreated systems lies in between the proportions reported in two similar reviews (23.9% [12] and 69.7% [11]). This may be because both of those reviews had narrower scope and included grey literature and thus are likely to have captured different outbreaks; the former studied Canada Communicable Disease Reports, ProMED-mail and Google grey literature, while the latter reviewed only the limited-access, likely non-English NWDOS system data for four countries.

The descending order in outbreak frequency for untreated, disinfection-only, and disinfection -and-filtration system outbreaks could arise from the relative extent of treatment applied and/or from the relative size of population served by each category, which likely changed substantively during the period analyzed in this review for example with the conversion of untreated systems to treated systems in much of the developed world. For example, many U.S. cities implemented filtration and chlorination treatment in the early 20th century [34]. This trend towards decreasing untreated system outbreaks (both surface water and groundwater) starting in the 1920s, excluding the low-reporting 1940s–1960s period, is supported by Figure 4a. The decline in unknown type treatment attribution beyond the 1980s could suggest improved DWDO reporting in developed countries (host to almost all identified outbreaks) associated with the adoptions of NWDOS by several European countries.

The increase in groundwater disinfection-only system outbreak frequency (Figure 4a) and the slight increase in log_10_ decadal case count for such outbreaks (Figure 4b) from the 1970s suggest the need for enhanced treatment and oversight of such systems. This contrasts the decrease in log_10_ decadal case count for surface water outbreaks of all five treatment types from the 1990s–on (which may further reflect the success of the aforementioned surface water-focused policies in reducing surface water DWDO disease burden). The increase in groundwater disinfection-only outbreak log_10_ decadal case count implies that the widespread approach of permitting disinfection-only groundwater systems may be insufficient. However, it is also possible that the increase derives from improved outbreak reporting, which could also explain other increasing frequency and case count trends.

The case counts of disinfection-only and disinfection-and-filtration source outbreaks are significantly (*p* < 0.0001 for both) higher than those for untreated source outbreaks. This may arise from untreated water sources being smaller and/or better-protected (e.g., with better siting) than the other two source types. In the latter case, if disinfection or one or both of disinfection and filtration failed consumers could be exposed to a higher risk source (more conducive to higher case count) as compared to typical untreated sources.

#### 4.2.6. Associated Deficiencies

Outbreaks associated with deficiencies in the realms of catchment or treatment are recorded more frequently (1.7 and 1.4 times, respectively) than outbreaks associated with distribution realm-associated deficiencies, a finding confirmed in an E.U. public drinking water supply-focused, 1990–2005 online DWDO literature review and analysis [35]. In that study, catchment and treatment realm-associated deficiencies were each (not necessarily singularly) recorded for 41 of the total 61 outbreaks, making them far more frequent than distribution realm-associated deficiencies, recorded for 19 outbreaks. However, although distribution realm-associated deficiencies are less frequent than those of the other realms, their outbreak case counts are significantly higher than those for outbreaks associated with the realms of catchment and treatment (*p* < 0.0001 and 0.0007, respectively). This could be due to increased ease of associating all ill patients with the same (more-localized) exposure in a more limited number of jurisdictions in distribution realm-associated deficiency outbreaks, increasing the probability of successful case reporting and attribution to a single outbreak.

There was a relatively high number of groundwater catchment realm-associated deficiency outbreaks in the 1920s and 1930s (Figure 5a) compared to the groundwater outbreaks associated with the other four deficiency realms. This may be explained by lower wastewater treatment capabilities during those decades. For example, with fewer U.S. cities implementing primary sewage treatment and sewage chlorination than water treatment by that time [34], sewage infiltration into (often untreated) water supplies might have presented a greater public health threat than treatment or distribution realm-associated deficiencies.

The decreasing frequency (and log_10_ decadal case count, Figure 5b) of treatment realm-associated deficiency outbreaks from the 1980s noted for surface water (and less so for groundwater) systems, consistent with the statistically-significant decrease (apparent for surface water outbreaks after introduction of the SWTR in 1989) in annual proportion of deficiencies related to treatment for the U.S. from 1971 to 2006 described in a 2010 review [31], could reflect improved treatment, especially in the U.S, the E.U., Australia and Canada. In the latter three, filtration of surface water is not required but rather recommended, with requirements for filtration existing at more local jurisdictional scales, e.g., in the Canadian provinces of Nova Scotia, Quebec, Ontario, Saskatchewan and Alberta [36]. In contrast, broadly steady outbreak frequency and log_10_ decadal case count for outbreaks with catchment or distribution realm-associated deficiencies in both surface water and groundwater systems since the 1980s (also consistent with the 2010 U.S. review’s determination of lack of statistically-significant change in annual proportion of distribution system deficiencies [31]) reflect the need to similarly strengthen oversight of those two realms. This could potentially be achieved through application of an integrated approach such as the Water Safety Plan, crafted and shown to successfully evaluate and manage risk from the catchment to the consumer (leading to statistically significant reductions in water supply contamination and waterborne disease in implementing areas in Iceland [37]).

### 4.3. Limitations

One person conducted the literature review and data extraction. The review was largely confined to peer-reviewed as well as English-language sources (found within four search databases that might not include all relevant literature), given that the search process did not target NWDOS or other grey literature and did not allow for translation of all non-English sources’ files. Examples of outbreaks within such grey literature not included in this review are the 175 NWDOS-based outbreaks from 1998 to 2012 for four Nordic countries captured within the web-based questionnaires used in [11], of which at most 17 outbreaks were reviewed in this study.

Issues of validity arose when sources’ authors considered outbreaks as occurring due to a single deficiency and attributed all cases to that deficiency, when in fact many such outbreaks should have been recorded as having multiple deficiencies. The tendency of outbreaks to derive from multiple deficiencies is exhibited in a similar review [32], which found a mean of 3.25 deficiencies per outbreak among the 61 outbreaks studied. The fault-tree weighting approach within that study, using a three-person consensus to discern the proportional contribution of deficiencies to outbreaks, shows promise in addressing the multiple-attribution limitation but was not applied in this research.

## 5. Conclusions

Data for 1519 drinking water disease outbreaks were collated from 475 primarily English-language, peer-reviewed literature sources. The contribution of 25 U.S. and Canada NWDOS-type review sources of over two-thirds of all identified outbreaks explains those sources’ strong influence on temporal and geographic outbreak frequencies (which were low for developing countries). Population-weighted outbreak frequency and total case count data suggest that DWDO reporting systems in Northern European countries are similar in strength to that of the U.S. The attributions of outbreaks to various causative pathogens, water system types, and other outbreak characteristics are comparable with those within other similar DWDO reviews, such as the high (decreasing) unknown etiology proportion, the high relative frequency of groundwater outbreaks relative to surface water ones, the near-majority (also decreasing) untreated system proportion, and the high frequencies of catchment and treatment realm-associated deficiencies relative to distribution realm-associated deficiencies. Logic concerning differences in supplied population size, number of treatment steps and other factors likely drive differences in attribute frequencies and most of the statistically-significant differences (based on Bonferroni-adjusted *p*-values) noted among case counts and attack rates by outbreak attribute, e.g., the case counts of outbreaks associated with distribution realm deficiencies are significantly higher than those of outbreaks associated with deficiencies in the realms of catchment and treatment (*p* < 0.0001 and 0.0007, respectively). While outbreak frequency and case count decreases can be associated with implementation of U.S. and European drinking water regulations, more systematic drinking water management (such as implementation of Water Safety Plans) would help address the remaining burden of disease attributed to the catchment and distribution realms and to groundwater viral and disinfection-only system outbreaks.

## Figures and Tables

**Table 1:** Outbreak attributes recorded for drinking water disease outbreaks.

Content Type	Attributes Recorded
Temporal	Year(s) and month(s) of outbreak duration
Geographic	Urban/rural status ^1^; national and subnational (e.g., state or province) jurisdiction
Pathogen	Outbreak-associated pathogen type and genus (including species/strain) ^2^
Water System	Source type/subtype and water treatment type ^3^
Deficiency	Realm and type ^4^
Disease Burden	Cases, hospitalizations, and deaths; positive stool counts or serology
Epidemiological	Attack rate ^5^

^1^ Outbreaks were classified as rural or urban as reported by source authors. When lacking, classification was performed by the first author based on how the outbreak-associated site corresponded with the U.S. Census definition of urban place as having at least 2500 residents [17], with designation as “Unknown” made when necessary information was unavailable; ^2^ Pathogen type classifications are viral, protozoan, bacterial, multiple, suspected (as labeled by source authors) or unknown; ^3^ Source type classifications are surface water, groundwater or unknown; source subtype classifications are river, lake, reservoir, well, spring, unknown, and the less frequent types storage tank, cistern, infiltration gallery, and rainwater collection; water treatment type classifications are untreated, disinfection-only (including chlorination, ultraviolet radiation and ozonation), disinfection-and-filtration, unknown, and the less frequent types (grouped as “other treatment”) filtration-only, bankside filtration-and-chlorination, reverse osmosis-only, and other undescribed treated; ^4^ Deficiency realm classifications are untreated surface water and groundwater (CDCS definitions), catchment, treatment, distribution, management, multiple realms and unknown; deficiency type classifications include specific faults within the catchment, treatment, distribution and management realms as described in Section 3.2.6; ^5^ The attack rate (*i.e.*, number of cases divided by affected population) recorded uses the water system-supplied population as the denominator.

**Table 2:** Outbreak health data by decade.

Decade	Median Case Count (*n*)	Total Case Count	Median Attack Rate (*n*)
Pre-1920	303 (14)	30,330	N/A (0)
1920s	27 (263)	95,610	5.3% (9)
1930s	23 (178)	46,179	10.5% (13)
1940s	370 (36)	71,839	26.4% (4)
1950s	71 (39)	95,094	8.7% (13)
1960s	170 (25)	30,732	11.3% (11)
1970s	59 (212)	77,585	36.2% (18)
1980s	59 (323)	149,486	28.0% (51)
1990s	63 (241)	641,243	28.0% (45)
2000+	71 (188)	112,980	12.5% (50)

**Table 3:** Outbreak health data by country (top nine countries).

Country ^1^	Median Case Count (*n*)	Population-Weighted Total Case Count (and Outbreak Frequency, both per 100,000 People)	Median Attack Rate (*n*)
U.S.	37 (1070)	391 (0.47)	24.0% (83)
U.K.	62 (131)	64 (0.23)	13.5% (33)
Canada	40 (106)	216 (0.43)	26.0% (14)
India	265 (46)	25 (0.01)	5.4% (22)
Sweden	529 (16)	353 (0.19)	68.4% (5)
France	782 (12)	19 (0.02)	15.7% (10)
Turkey	42 (10)	2 (0.02)	12.7% (3)
Spain	100 (10)	19 (0.03)	28.9% (4)
Finland	1350 (10)	475 (0.21)	18.7% (7)

^1^ At the subnational level, within the U.K. 87, 12, eight and four outbreaks are attributed to England, Scotland, Wales and Northern Ireland, respectively (with the rest classified as “None Given” or “Other” for territories). Within Canada, the provinces of Quebec, British Columbia and Ontario are associated with 40, 23 and 21 outbreaks, respectively (79.2% of the total for Canada).

**Table 4:** Outbreak health data by U.S. state (top nine states).

State	Median Case Count (*n*)	Population-Weighted Total Case Count and Outbreak Frequency, per 100,000 People
Pennsylvania	30 (170)	254 (1.43)
Colorado	28 (72)	457 (2.49)
New York	50 (72)	342 (0.41)
California	53 (40)	209 (0.17)
Illinois	79 (35)	96 (0.31)
Florida	5 (34)	53 (0.35)
Indiana	30 (34)	586 (0.62)
Washington	46 (33)	143 (0.80)
Oregon	59 (31)	2687 (1.18)

**Table 5:** Outbreak health data by urban/rural status.

Urban/Rural Status	Median Case Count (*n*)	Median Attack Rate (*n*)
Urban	170 (417)	12.6% (97)
Rural	37 (610)	28.8% (101)
Unknown	38 (492)	23.0% (16)

**Table 6:** Outbreak health data by pathogen type (in bold) and genus.

Pathogen Type/Genus ^1^	Median Case Count (*n*)	Median Attack Rate (*n*)
**Bacterial**	**69 (282)**	**23.1% (58)**
*Campylobacter*	50 (71)	29.1% (24)
*Salmonella*	68 (69)	13.1% (11)
*Shigella*	115 (64)	36.1% (8)
*E. coli* (Enterovirulent)	22 (23)	16.5% (5)
**Protozoan**	**50 (254)**	**16.7% (55)**
*Giardia*	32 (153)	17.0% (26)
*Cryptosporidium*	72 (75)	0.6% (11)
**Viral**	**97 (149)**	**8.7% (42)**
Hepatitis	50 (86)	7.9% (30)
Norovirus	180 (56)	41.0% (11)
**Multiple Types**	**200 (25)**	**44.5% (10)**
**Unknown Type ^2^**	**37 (809)**	**27.3% (49)**

^1^ The frequencies of outbreaks’ other associated pathogens (for which statistics are not given), listed in descending order, are as follows: *Vibrio cholerae* (*n* = 24), *Entamoeba histolytica* and *Francisella tularensis* (*n* = 8 each), *Leptospira* spp. and *Cyclospora* spp. (*n* = 4 each), *Blastocystis hominis*, *Toxoplasma gondii* and Rotavirus (*n* = 3 each), *Yersinia* spp., *Clostridium perfringens* and *Enterovirus C*, *i.e.*, poliovirus (*n* = 2 each), and *Plesiomonas shigelloides*, *Providencia* spp., *Streptobacillus moniliformis*, *Burkholderia pseudomallei* and *Enterovirus echovirus* (*n* = 1 each). The pathogen genus rows do not comprise all of the outbreaks within each pathogen type, e.g., the 254 only-Protozoan outbreaks include mixed-protozoan outbreaks not counted within 153 and 75 only-*Giardia* and only-*Cryptosporidium* outbreaks, respectively; ^2^ Suspected and unknown pathogen type-associated outbreaks are classified together as “Unknown Type”.

**Table 7:** Outbreak health data by source type (in bold) and subtype.

Source Type/Subtype ^1^	Median Case Count (*n*)	Median Attack Rate (*n*)
**Surface Water**	**53 (450)**	**22.4% (70)**
River	40 (266)	22.0% (47)
Lake	82 (70)	6.7% (6)
Reservoir	151 (38)	10.2% (6)
**Groundwater**	**41 (852)**	**24.0% (113)**
Well	44 (609)	24.2% (79)
Spring	36 (164)	14.0% (27)
**Mixed Types**	**67 (50)**	**6.1% (10)**
**Unknown**	**80 (167)**	**10.0% (21)**

^1^ Statistics are not given for the source subtypes storage tank, rainwater collection, infiltration gallery and cistern, with six, four, three and one outbreak(s) recorded, respectively.

**Table 8:** Outbreak health data by water treatment type.

Water Treatment Type	Median Case Count (*n*)	Median Attack Rate (*n*)
Untreated	31 (704)	25.6% (70)
Disinfection-Only	94 (271)	16.7% (74)
Disinfection-and-Filtration	108 (117)	28.2% (32)
Other Treatment ^1^	207 (72)	5.1% (21)
Unknown Treatment	53 (355)	13.9% (17)

^1^ Includes the water treatment types other undescribed treated, filtration-only, bankside filtration-and- chlorination, and reverse osmosis-only with 47, 21, three and one outbreak(s) recorded, respectively.

**Table 9:** Outbreak health data by deficiency realm (in bold) and type.

Deficiency Realm/Type ^1^	Median Case Count (*n*)	Median Attack Rate (*n*)
**Catchment**	**37 (357)**	**28.4% (54)**
Sewage Contamination	92 (86)	50.5% (24)
Septic Tank Runoff	75 (14)	7.7% (3)
Animal Watershed Contamination	32 (16)	15.8% (2)
Rainfall/Runoff	19 (11)	44.0% (2)
Source Siting/Enclosure	48 (27)	14.3% (2)
**Treatment**	**44 (289)**	**17.2% (21)**
Chlorination	30 (91)	14.3% (10)
Filtration	78 (33)	23.0% (6)
**Distribution**	**85 (214)**	**14.2% (40)**
Cross-Connection	47 (76)	13.9% (5)
Backflow	41 (18)	24.4% (5)
Storage	182 (18)	29.3% (6)
**Untreated Surface Water**	**32 (30)**	**N/A (0)**
**Untreated Groundwater**	**33 (174)**	**69.5% (2)**
**Multiple Realms**	**126 (280)**	**16.7% (81)**
**Unknown**	**31 (173)**	**25.8% (16)**

^1^ Statistics are not given for the deficiency realm “Management”, with two outbreaks recorded, or for the other catchment, treatment, distribution and management realm-associated deficiency types.

## Abbreviations

The following abbreviations are used in this manuscript:

CDCS	(U.S.) Centers for Disease Control Summary
DWDO	Drinking Water Disease Outbreak
ESPM	Environmental Sciences and Pollution Management
EU	European Union
GW	Groundwater
NORS	(U.S.) National Outbreak Reporting System
NWDOS	National Waterborne Disease Outbreak Surveillance
PDF	Portable Document Format
SWTR	Surface Water Treatment Rule
WHO	World Health Organization
WoS	Web of Science

## References

[1] Bartram J., Hunter P., Bartram J., Baum R., Coclanis P., Gute D., Kay D., McFadyen S., Pond K., Robertson W., Rouse M. (2015). Bradley classification of disease transmission routes for water-related hazards. Routledge Handbook of Water and Health.

[2] Tirado C., Schmidt K. (2001). World Health Organization Surveillance Program for Control of Foodborne Infections and Intoxications: Preliminary Results and Trends across Greater Europe. J. Infect..

[3] MacKenzie W.R., Hoxie N.J., Proctor M.E., Gradus S., Blair K.A., Peterson D.E., Kazmierczak J.J., Addiss D.G., Fox K.R., Rose J.B. (1994). A massive outbreak in Milwaukee of *Cryptosporidium* infection transmitted through the public water-supply. N. Engl. J. Med..

[4] Medema G.J., Payment P., Dufour A., Robertson W., Waite M., Hunter P., Kirby R., Andersson Y., Dufour A., Snozzi M., Koster W., Bartram J., Ronchi E., Fewtrell L. (2003). Safe drinking water: An ongoing challenge. Assessing Microbial Safety of Drinking Water Improving Approaches and Methods: Improving Approaches and Methods.

[5] On the Mode of Communication of Cholera. http://www.ph.ucla.edu/epi/snow/snowbook.html.

[6] National Outbreak Reporting System (NORS, Water). http://www.cdc.gov/healthywater/statistics/wbdoss/nors/.

[7] National Research Council (2004). Indicators for Waterborne Pathogens.

[8] Kramer M.H., Quade G., Hartemann P., Exner M. (2001). Waterborne diseases in Europe: 1986–1996. J. Am. Water Works Assoc..

[9] Bartram J., Thyssen N., Gowers A., Pond K., Lack T. (2002). Water and Health in Europe: A Joint Report from the European Environment Agency and WHO Regional Office for Europe.

[10] Risebro H.L., Hunter P.R. (2007). Surveillance of waterborne disease in European member states: A qualitative study. J. Water Health.

[11] Guzman-Herrador B., Carlander A., Ethelberg S., de Blasio B.F., Kuusi M., Lund V., Löfdahl M., MacDonald E., Nichols G., Schönning C. (2015). Waterborne outbreaks in the Nordic countries, 1998 to 2012. Eurosurveillance.

[12] Pons W., Young I., Truong J., Jones-Bitton A., McEwen S., Pintar K., Papadopoulos A. (2015). A systematic review of waterborne disease outbreaks associated with small non-community drinking water systems in Canada and the United States. PLoS ONE.

[13] Schuster C.J., Ellis A.G., Robertson W.J., Charron D.F., Aramini J.J., Marshall B.J., Medeiros D.T. (2005). Infectious disease outbreaks related to drinking water in Canada, 1974–2001. Can. J. Public Health.

[14] Hunter P.R., Syed Q. (2001). Community surveys of self-reported diarrhoea can dramatically overestimate the size of outbreaks of waterborne cryptosporidiosis. Water Sci. Technol..

[15] Brunkard J.M., Ailes E., Roberts V.A., Hill V., Hilborn E.D., Craun G.F., Rajasingham A., Kahler A., Garrison L., Hicks L. (2011). Surveillance for waterborne disease outbreaks associated with drinking water—United States, 2007–2008. Morb. Mortal. Wkly. Rep..

[16] Tillett H.E., de Louvois J., Wall P.G. (1998). Surveillance of outbreaks of waterborne infectious disease: Categorizing levels of evidence. Epidemiol. Infect..

[17] United States Census Bureau (2013). Chapter 12: The Urban and Rural Classifications. Geographic Areas Reference Manual.

[18] United States Census Bureau Population Estimates. https://www.census.gov/popest/data/state/asrh/1980s/80s_st_totals.html.

[19] Demographic Yearbook: Population Censuses’ Datasets (1995–Present). http://unstats.un.org/unsd/demographic/products/dyb/dybcensusdata.htm.

[20] Simple and Sophisticated Bonferroni Adjustment. http://privatewww.essex.ac.uk/~scholp/bonferroni.htm.

[21] Engineering Statistics Handbook 7.1.6. What Are the Outliers in the Data?. http://www.itl.nist.gov/div898/handbook/prc/section1/prc16.htm.

[22] Gorman A.E., Wolman A. (1939). Water-borne outbreaks in the United States and Canada and their significance. J. Am. Water Works Assoc..

[23] Global Launch of the Human Development Report. http://hdr.undp.org/en/2015-report.

[24] Regli S., Rose J.B., Haas C.N., Gerba C.P. (1991). Modeling the risk from giardia and viruses in drinking water. J. Am. Water Works Assoc..

[25] Andersson Y., Bohan P., Fewtrell L., Bartram J. (2001). Disease surveillance and waterborne outbreaks. Water Quality Guidelines, Standards and Health: Assessment of Risk and Risk Management for Water-Related Infectious Disease.

[26] Progress on Sanitation and Drinking Water: 2015 Update and MDG Assessment. http://www.wssinfo.org/fileadmin/user_upload/resources/JMP-Update-report-2015_English.pdf.

[27] Rooney R.M., Bartram J.K., Cramer E.H., Mantha S., Nichols G., Suraj R., Todd E.C.D. (2004). A review of outbreaks of waterborne disease associated with ships: Evidence for risk management. Public Health Rep..

[28] St. Louis M.E. (1988). Water-Related Disease Outbreaks, 1985. Morb. Mortal. Wkly. Rep..

[29] Food and Waterborne Disease. http://www.floridahealth.gov/diseases-and-conditions/food-and-waterborne-disease/index.html.

[30] Sinclair R.G., Jones E.L., Gerba C.P. (2009). Viruses in recreational water-borne disease outbreaks: A review. J. Appl. Microbiol..

[31] Craun G.F., Brunkard J.M., Yoder J.S., Roberts V.A., Carpenter J., Wade T., Calderon R.L., Roberts J.M., Beach M.J., Roy S.L. (2010). Causes of outbreaks associated with drinking water in the United States from 1971 to 2006. Clin. Microbiol. Rev..

[32] Council Directive 75/440/EEC. http://www.eea.europa.eu/policy-documents/council-directive-75-440-eec.

[33] Craun G.F. (2012). The importance of waterborne disease outbreak surveillance in the United States. Ann. Ist. Super. Sanita.

[34] Cutler D., Miller G. The Role of Public Health Improvements in Health Advancements: The 20th Century United States. http://www.nber.org/papers/w10511.pdf.

[35] Risebro H.L., Doria M.F., Andersson Y., Medema G., Osborn K., Schlosser O., Hunter P.R. (2007). Fault tree analysis of the causes of waterborne outbreaks. J. Water Health.

[36] Boyd D.R. The Water We Drink: An International Comparison of Drinking Water Quality Standards and Guidelines. http://www.davidsuzuki.org/publications/downloads/2006/DSF-HEHC-water-web.pdf.

[37] Gunnarsdóttir M.J. (2012). Safe Drinking Water: Experience with Water Safety Plans and Assessment of Risk Factors in Water Supply. Ph.D. Thesis.

